# Gamma Delta T Cells and Their Pathogenic Role in Psoriasis

**DOI:** 10.3389/fimmu.2021.627139

**Published:** 2021-02-25

**Authors:** Cong Qi, Yazhuo Wang, Ping Li, Jingxia Zhao

**Affiliations:** ^1^ Beijing Hospital of Traditional Chinese Medicine, Capital Medical University, Beijing, China; ^2^ Beijing Key Laboratory of Clinic and Basic Research with Traditional Chinese Medicine on Psoriasis, Beijing Institute of Traditional Chinese Medicine, Beijing, China

**Keywords:** psoriasis, γδT, IL-17, skin inflammation, biological function

## Abstract

γδT cells are an unconventional population of T lymphocytes that play an indispensable role in host defense, immune surveillance, and homeostasis of the immune system. They display unique developmental, distributional, and functional patterns and rapidly respond to various insults and contribute to diverse diseases. Although γδT cells make up only a small portion of the total T cell pool, emerging evidence suggest that aberrantly activated γδT cells may play a role in the pathogenesis of psoriasis. Dermal γδT cells are the major IL-17-producing cells in the skin that respond to IL-23 stimulation. Furthermore, γδT cells exhibit memory-cell-like characteristics that mediate repeated episodes of psoriatic inflammation. This review discusses the differentiation, development, distribution, and biological function of γδT cells and the mechanisms by which they contribute to psoriasis. Potential therapeutic approaches targeting these cells in psoriasis have also been detailed.

## Introduction

Gamma delta T cells (γδ T cells) are T cells that have a distinctive T-cell receptor (TCR) on their surface. Most T cells are αβ (alpha beta) T cells with TCR composed of two glycoprotein chains called α (alpha) and β (beta) TCR chains. In contrast, gamma delta (γδ) T cells have a TCR that is made up of one γ (gamma) chain and one δ (delta) chain (1). This group of T cells is usually less common than αβ T cells, but significantly enriched in mucosal and epithelial sites, such as the skin and respiratory, digestive, and reproductive tracts. γδT cells are major histocompatibility complex (MHC)-unrestricted innate-like lymphocytes with more unique antigen receptors compared to αβT cells (2). They produce cytokines such as IL-17/IFN-γ/IL-22 (3–5). Although they constitute a small portion of the total T cell pool, γδT cells bridge the innate and adaptive immune system and contribute to various physiological and pathological processes (2). Relative to αβT cells, γδT cells have been less studied and characterized. It is becoming clear that γδT cells are heterogeneous populations of cells with multifunctional capacities in repairing host tissue (6), pathogen clearance (7), tumor surveillance (8, 9), and proinflammatory effects (10).

Psoriasis is a chronic inflammatory skin disease with an autoimmune component and a strong genetic basis. Plaque psoriasis is characterized by well-defined, raised, chronic erythematous plaques with silver patches observed commonly in the elbows, knees, scalp, umbilicus, and lumbar area (11–13). The worldwide reported prevalence of psoriasis ranges from 0.09% to 11.43% and results in a severe economic burden to patients and a significant challenge to public health (14, 15). Multiple comorbidities and other autoimmune disorders have been correlated with psoriasis, which includes arthritis, cardiovascular disease, obesity, diabetes mellitus, and inflammatory bowel disease, indicate common cellular mediators that drive the pathogenesis of these diseases (16). Increasing evidence has demonstrated that aberrantly activated γδT cells may direct the pathogenesis of autoimmune disorders, such as psoriasis (17–19). To understand what they do in psoriasis, it is important to understand their the differentiation, development, distribution, and biological function.

In this review, we expound on the properties of γδT cells and review the effects of γδT cells in psoriasis. We hope that this review provides insights into its pathogenesis, especially in disease recurrence, and sheds light on potentially novel therapies targeting γδ T cell function.

## Differentiation and Development of γδT Cells in the Thymus

**Figure 1:**
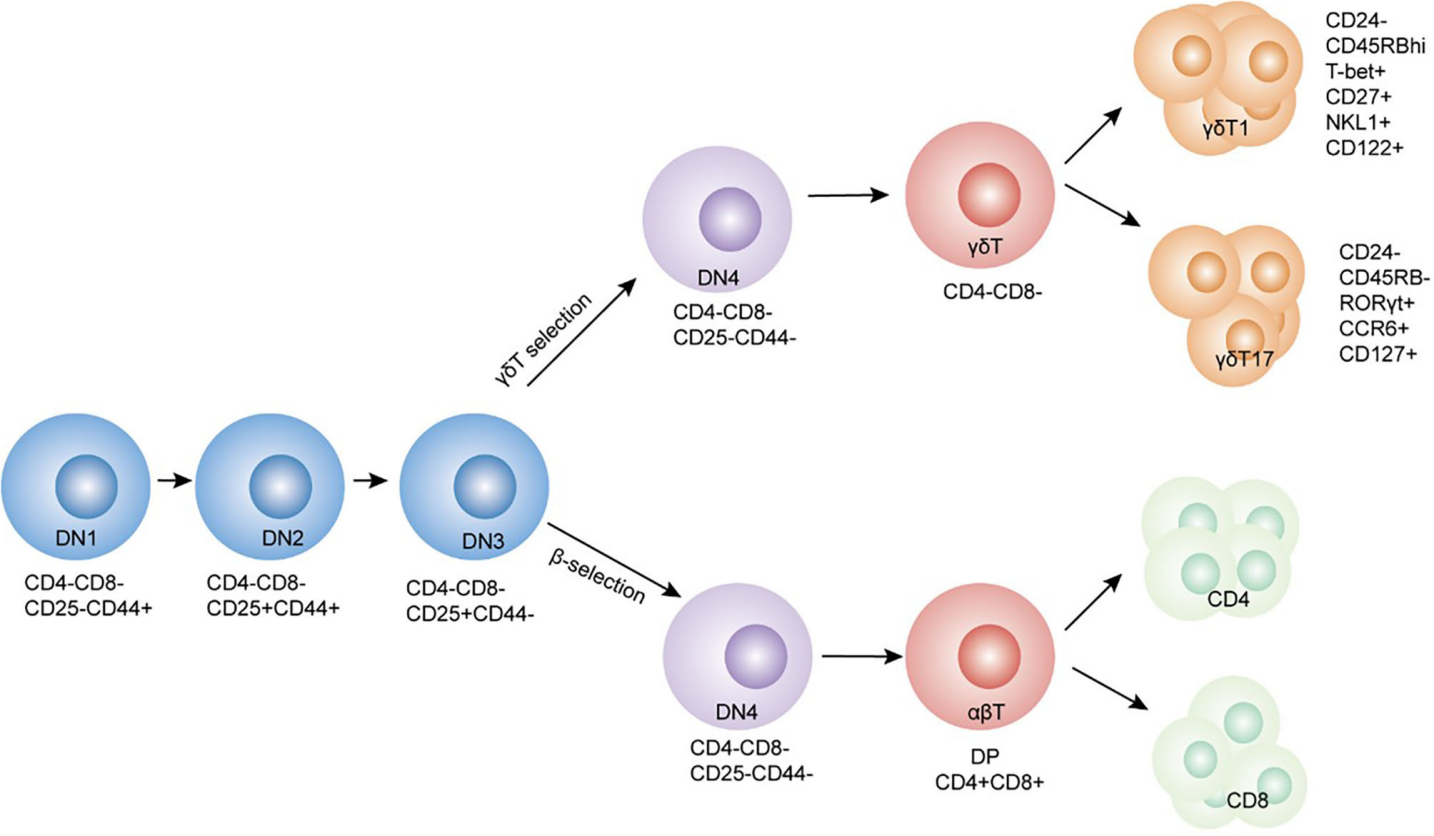
Schematic depicting the development of γδ and αβ T cells in the thymus. γδ and αβ cells develop from the same ancestral DN (CD4-CD8-) cells. Lineage changes in γδ and αβ cells mainly occur at the DN3 stage. Cell surface markers and transcription factors of the cells have been labeled alongside.

γδT cells were first discovered and reported 30 years ago during the manufacture of antibodies using the *TCRγ* gene sequence (20). αβT and γδT cell lineages originate from common T precursor cells that lack CD4 and CD8 coreceptors (CD4-CD8-), also known as double-negative (DN) thymocytes. Based on their differential CD44 and CD25 expression, DN cells can be further subdivided into DN1 (CD44+CD25-), DN2 (CD44+CD25+), DN3 (CD44-CD25+), and DN4 (CD44-CD25-) stages, as indicated in **Figure 1**. Clonal assays for determining DNT cell progenitors permit the identification of the branch-point of αβT and γδT cell lineages at the late DN2 to DN3 developmental stages (21, 22). DN3 is the critical selection stage that determines the fate of γδ or αβ cell lineages (22). Rearrangements at the *Tcrd*, *Tcrg*, and *Tcrb* loci are initiated at the DN2 stage, and αβ and γδ lineage divergence occur at the DN3 stage (23, 24). Successful rearrangement of the TCRβ chain is achieved with the assembly of the constant pTα and CD3 subunits to form the pre-TCR complex. Commitment to the αβT cell lineage and differentiation of DN3 cells into DN4 (CD44-CD25-) cells transpires in a ligand-independent manner. This process is termed β-selection and is a checkpoint for the generation of a functional TCRβ chain (25, 26). TCRγ and δ chains rearrange during the DN stages and express γδTCR/CD3 on the plasma membrane. ‘γδ selection’ is associated with increased extracellular signal-related kinase 1/2(ERK1/2) phosphorylation and early growth response gene (Egr) protein expression. Ectopic expression of Egr proteins promotes the selection of the γδ T cells. Inhibitor of DNA binding 3 (Id3) is an essential target by which Egr proteins regulate αβ/γδ lineages (26–29).

What determines cell fate specification and differentiation from precursors to αβT or γδT lineage? Two models have been proposed, an instructive model and a stochastic model. In the instructive model, pre-TCR or γδ TCR signaling intensities decide the fate of αβT/γδT cell lineage (30). The instructive model is based on several lines of evidence showing that the strong TCR signals are inclined to γδT cells, while the relatively weak TCR signals are inclined to generate abundant αβT lineage cells (27, 31, 32). The stronger signals that promote adoption of the γδ-fate involve activation of the ERK-Egr-Id3 pathway (29, 33). Sang-Yun et al. demonstrated that ERK signaling promotes γδT cell maturation. ERK signaling that promotes γδT cell fate depends not only on conventional substrate targeting through the D-domain but also through an alternate mode of ERK action mediated by its DBP. This induces molecular effectors responsible for the execution of ERK-mediated developmental outcomes post-transcriptionally (34). E proteins are helix-loop-helix transcription factors that bind DNA at E-box motifs (CANNTG). It acts as a downstream focal point for TCR and plays an essential role in thymocyte development (35). Strong TCR signals could selectively restrain αβT cell development by phenocopying E protein insufficiency and increasing ERK activation. This induces early growth response (EGR1, EGR3) transcription factors and targets DNA-binding inhibitors (ID3). ID3 has been shown to interact with and suppress E protein targets (33, 36, 37).

Under stochastic conditions, other signals dominate this differentiation before TCR expression, hence pre-committing cell fate and allowing them to mature further. Increasing evidence has presented that progenitor T cells are heterogeneous in their developmental potential prior to TCR gene rearrangement. Their development potential has been associated with IL-7R expression (pre-T cells) and was independent of TCR-mediated signals (30). High mobility group box transcription factor 13 (Sox13) that modulate Wnt/TCF1 signaling has also been reported to regulate the T cell-fate decision process, while Sox13 expression has been shown to promote γδT cell development and restrain αβT cell development (38, 39). Nevertheless, γδT cell development has been observed in Sox13-deficient mice, suggesting that it is dispensable for γδT cell development. This is contrary to what has been suggested in the stochastic model (38).

## Distribution of γδT

### Human γδT Cells

Humans γδT cells can be distinguished based on δ chain expression, which includes the Vδ1, Vδ2, and Vδ3 subtypes (40) (**Table 1**). Vδ1 cells are mainly found in the gut epithelium, skin, spleen, and liver, and are involved in maintaining epithelial tissue integrity. They constitute approximately 30% of the γδT cells in the peripheral blood (PB). Typically, the Vδ1 chain is associated with different VγI family members (Vγ2/3/4/5/8/9) (41–43). Vδ1 cells exert their effector function through TCR recognition of stress molecules on epithelial cells. Furthermore, Vδ1 cells express natural killer receptors (NKG2C, NKG2D, NKp30), Toll-like receptors, CD8, and the β-glucan receptor, dectin-1 (44–48). Activated Vδ1T cells release IL-10, IL-2, IL-4, IL-17, IFN-γ, TNF-α, and chemokines (CCL3, CCL4, and CCL5). Vδ1T cells play an essential role in maintaining barrier tissue integrity and establishing antiviral immunity (49–51). Studies have demonstrated that Vδ1 cells are involved in several diseases, such as malaria (52, 53), human immune deficiency virus (HIV) (54, 55), cytomegalovirus (CMV) (56), inflammatory bowel disease, and Crohn’s disease by exerting their cytotoxic effects and secreting cytokines (57). Notably, activated Vδ1T cells recognize B7-H6 *via* NKp30. B7-H6 is a B7 family member exclusively expressed on tumor cells and is involved in the antitumor effect (58).

**Table 1:** Characteristics of human and murine γδT cell subsets.

	Classify	Common pairs	Tissue resident	Production of cytokines
Human	Vδ1	Vγ2^+^/3^+^/4^+^/5^+^/8^+^/9^+^	gut, skin, liver PB (peripheral blood)	IL-10,IL-2,IL-4,IL-17,IFN-γ, TNF-α
	Vδ2	Vγ9^-^/9^+^	PB, skin	IFN-γ,TNF-α,IL-17,IL-21,IL-24
	Vδ3	Vγ2^+^/3^+^/4^+^	PB, liver	IL-10,IL-4,IL-17,IFN-γ,TNF-α
Murine	Vγ1	Vγ6.3/6.4	skin, lung, colon, liver, PB	IL-4,IFN-γ
	Vγ2	Vδ4	skin, lung, colon, liver, PB	IL-17
	Vγ3	Vδ1	skin	?
	Vγ4	Vδ4	skin, lung, colon, liver, PB, joint	IL-17,IFN-γ
	Vγ5	Vδ1	skin, liver	IL-17,IFN-γ
	Vγ6	Vδ1	genital tract, togue, lung, colon, skin, adipose tissue	IL-17,IFN-γ,IL-22
	Vγ7	Vδ4/5/6	IEL (Intraepithelial lymphocytes)	IFN-γ

Heilig and Tonegawa nomenclature used for classification.

Vδ2T cells are primarily distributed in the blood and the lymphoid system and are the main subset found in healthy humans. It accounts for 50%–90% of the γδT cell population in peripheral blood (59). Vδ2T cells are divided into the innate-like (Vγ9+) and adaptive (Vγ9-) subsets, with the majority of Vδ2T cells being Vδ2Vγ9+T cells (60). Vδ2Vγ9+T cells are responsive to cytokines, such as CCR1, CCR2, CCR5, and CXCR6 ligands and IL-12, and produce proinflammatory factors, such as IFN-γ, TNF-α, IL-17, IL-21, and IL-24 (61, 62). Vδ2Vγ9+T cells can be divided into naive γδT (CD45RA+CD27+Vδ2Vγ9+), central memory γδT (TCM, CD45RA-CD27+Vδ2Vγ9), effector memory γδT (TEM, CD45RA-CD27-Vδ2 Vγ9+), and CD45RA+ effector memory γδT (TEMRA, CD45RA+CD27-Vδ2Vγ9+) based on their surface expression of CD45RA and CD27. Naive γδT cells comprise of the Vδ2Vγ9+T cell subset in the lymph nodes and express CCR7 and CD62L. However, CCR2, CCR5, CCR6, and CXCR3 are only expressed and activated in the presence of high concentrations of isopentenyl pyrophosphate (IPP) but do not produce IFN-γ. CM cells express CCR7 and CD62L and are activated at low IPP concentrations and produce some IFN-γ. TEM cells are present in the blood and inflammatory sites and are CCR7-CD62L-. However, they are positive for the chemokine receptors CCR2, CCR5, CCR6, and CXCR3. TEM cells secrete abundant IFN-γ and tumor necrosis factor-alpha (TNF-a) when activated with IPP+IL-2. TEMRA cells are CCR7-CD62L- but express CCR5 and CXCR3, and have a cytotoxic effect. TEMRA cells also secrete abundant perforin, granulysin, and N-a-benzyloxycarbonyl-L-lysine thiobenzyl ester (BLT)-esterase, but do not produce IFN-γ. In addition, they are terminally differentiated and are no longer able to respond to TCR stimulation, and have poor proliferative ability (63–66). Vδ2 specifically recognizes (E)-4-hydroxy-3-methyl-but-2-enyl pyrophosphate (HMB-PP) and isopentenyl pyrophosphate (IPP) and rapidly respond to exogenous infections or endogenous transformed cells (67, 68). Furthermore, activated Vδ2Vγ9+T cells acquire antigen-presenting cell (APC) characteristics and display a strong ability to secrete cytokines, such as Th1/Th2/Th17-type cytokines. These induce the maturation of dendritic cells (DCs) into APCs (69–71).

Vδ3T cells are the smallest subsets of the peripheral blood lymphocytes, accounting for 0.2% of circulating cells. They express CD56, NKG2D, CD28, HLA-DR, CD161, and T cell activation marker CD69, but not CD25, NKG2A, or NKG2C (72). Vδ3T cells are abundant in the liver and gut and are involved in chronic viral infections and leukemia (73, 74). Expanded Vδ3T cells only recognize CD1d and release Th1, Th2, and Th17 cytokines to induce the maturation of dendritic cells into APCs. They do not recognize CD1a, CD1b, or CD1c (72). Vδ3 T cells and B cells reciprocally regulate the expression of maturation markers, CD40, CD86, and HLA-DR, and promote IgM release by B cells (75).

Interestingly, Vδ4, Vδ6, Vδ7, and Vδ8 T cells have been observed in the PB of lymphoma patients, however, their roles are yet to be deciphered (76).

### Murine γδ T Cells

Murine γδT cells can be distinguished based on their γ chain expression. Two nomenclature methods have been commonly reported in the literature, i.e., the Heilig and Tonegawa, and the Garman classification (77, 78). This review uses the Heilig and Tonegawa nomenclature and is used for the Vγ1–Vγ7 subtypes (79) (**Table 1**).

The development of the γδT subsets begins during the fetal period. First are the Vγ5+cells that are produced between embryonic day13 (E13) to approximately E17, followed by Vγ6+ cells from E14 to around birth, and the last are the Vγ1+, Vγ2+, and Vγ4+ cells from E16 onward (25, 80, 81). Vγ5+ cells, also known as dendritic epidermal T cells (DETCs), are involved in innate body barrier defense. The increased expression of sphingosine-1-phosphate receptor 1 (S1P1), E and P selectin ligands, and chemokines CCR10 and CCR4 in mature Vγ5+ cells, and the decreased expression of CCR6, CCR9, CCR7, and CD62L allow the egression of Vγ5+ cells from the thymus to the epidermis (82, 83). In normal healthy skin, DETC secretes IL-15 and IGF-1 to maintain skin homeostasis and promote wound healing (84, 85). After skin trauma, DETCs undergo morphological changes accompanied by the upregulation of the activation marker, CD69. It then releases soluble factors that regulate various aspects of tissue repair (85). DETCs produce CCL3 and CCL4 chemokines that are important for macrophage homing. Furthermore, DETCs promotes macrophage recruitment by regulating hyaluronan production through DETC-derived keratinocyte growth factor (KGF) (86, 87). Vγ5+Vδ1+cells produce IFN-γ by activating the Egr3-mediated pathway while suppressing the γδT cell lineage factor, Sox13, and the RORγt transcription factor associated with IL-17 production (39). However, some studies have shown that DETCs produce IL-17, promote keratinocyte proliferation, and participate in skin inflammation (88).

The second γδT subsets produced are the Vγ6 cells. They pair with the Vδ1 subsets of γδ TCR (Vγ6Jγ1 and Vδ1Dδ2Jδ2) and migrate to the genital tract, tongue, lungs, peritoneal cavity (PEC), dermis, colon, and adipose tissues (89). Vγ6+Vδ1+ γδT cells that produce IL-17 and other effector molecules drive inflammation and tumor cell proliferation (90).

Typically, Vγ1+, Vγ2+, and Vγ4+ cells migrate to the dermis, lungs, colon, liver, and peripheral lymphoid organs (91). Both Vγ1+ and Vγ4+ cells can secrete IFN-γ, TNF-a, TGF-β, and IL-10 upon activation. However, Vγ1+γδT cells are predisposed to produced IL-4 and IL-5, while Vγ4+γδT cells preferentially produce IL-17 (92). Vγ1+γδT cells occur mainly in the form of Vγ1Vδ6.3/6.4 TCR cells and secrete IL-4 and IFN-γ (93). Upon acute infection with Coxsackievirus B3 (CVB3), Vγ1+γδT cells are the early and primary producers of IL-4 and play a protective role in CVB3 myocarditis (94). Vγ4+ γδT cells express high levels of Rorc, Sox13, Scart, Bclaf1, and Atf2 and secrete abundant levels of IL-17A and IL-17F (92) (95). IL-17A-producing Vγ4+γδT cells also express high levels of CCR6 on their surface and are chemoattracted by CCL20 that are secreted by keratinocytes to inflammatory sites, which in turn facilitates keratinocytes to secrete IL-1β and IL-23 (96). In addition, IL-17 secreted by Vγ4+γδT cells inhibits the production of IGF-1, thereby delaying skin wound healing (84, 97). Studies have shown that Vγ2+ T cells recruit neutrophils and aggravate liver fibrosis by secreting IL-17A (98, 99). It has also been demonstrated that Vγ7+T cells are the main components of the murine intestinal intraepithelial T cell compartment. Consequently, the selective maturation and expansion of Vγ7+T cells are driven by both Btnl1 and Btnl6 (100).

## Biological Effects of γδT Cells

γδT cells have strong plasticity and secrete different cytokines and chemokines. They exhibit diverse functions similar to Th1, Th2, Tregs, and Th17 cells in different microenvironments (2). Some γδ T cells generate growth factors such as VEGF, FGF-2, and IGF-1, suggesting that these cells have the capacity to maintain epithelial integrity and wound repair (101). Nonetheless, some γδ T cells have been reported to induce the production of antimicrobial peptides, including β-defensin 2, S100A7, and S100A8 in keratinocytes to exert a protective function in local epithelial defense (101). γδT cells secrete interleukin-10 (IL-10), control CD8+ T cell expansion, and regulate and reduce TNF-a secretion by activated CD8+ T cells (102). The role of IL-17-producing γδT cells has been investigated in various models of infection and autoimmunity (103, 104). IL-17-producing γδT cells robustly direct the recruitment of neutrophils and monocytes to increase the inflammatory response.

γδ T cells are involved in the regulation of macrophage homeostasis and recruitment. In patients suffering from listeriosis (a serious infection caused by the germ Listeria monocytogenes), γδT cells play a critical role in neutrophil replacement by producing chemokines such as macrophage chemoattractant protein1 (MCP-1) (105). Additional evidence has shown that γδT cells facilitate differentiation of the monocyte/macrophage lineage. Remarkably, monocytes differentiate into inflammatory macrophages during bacterial infections but fail to undergo maturation in mice lacking γδT cells (106). In contrast, the role of Vγ4 has been demonstrated to enhance macrophage activity and the production of specific pro-inflammatory and immunoregulatory cytokines by macrophages. Different subsets of γδT cells have opposing roles in macrophage homeostasis, indicating the complexity and plasticity of γδT cells (107). γδT cells present antigens to αβT cells, while Vδ2+ T cells display characteristics similar to professional APCs. Once activated, these cells efficiently process and present antigens and prime co-stimulatory signals for potent induction of αβT cell proliferation and differentiation (108). Receptors associated with DC, such as antigen presentation molecules (MHC class II), co-stimulatory receptors (CD40, CD80, and CD86), maturation markers (CD83), and adhesion receptors (CD11a, CD11b, CD11c, CD18, CD50, and CD54) have been found to be expressed on the surface of activated γδT cells (109, 110).

Activated γδT cells exhibit a broad range of cytotoxic activity, especially against a wide variety of tumor cells that utilize death receptor/ligand (Fas/Fas-ligand)-dependent and perforin/granzyme or granulysin-dependent pathways. Exogenous IL-18 promotes the expansion of γδT cells in human peripheral blood mononuclear cells (PBMCs) stimulated by Zoledronate (Zol) and IL-2 (109). The expansion of γδT cells is inhibited by neutralizing anti-IL-18 receptor antibodies, indicating that IL-18 efficiently promotes the expansion of γδT cells with potent antitumor activity (110). Furthermore, studies have shown that γδT cells directly kill activated hepatic stellate cells (HSCs) and increase NK cell-mediated cytotoxicity against activated HSCs in liver fibrosis (10).

γδT cells are highly efficient in promoting B cell maturation and producing IgM, IgG, and IgA antibodies. Vδ2Vγ9 T cells express IL-21R on their surface, which is enhanced upon HMB-PP induced irritation (111, 112). Activated Vδ2Vγ9T cells express CXCL13, CXCR5, and ICOS and upregulate the expression of B cell surface markers CD25, CD69, CD40, and CD86. This suggests that CXCR5+ Vδ2Vγ9 T cells are a distinct memory T cell subset with B cell helper function (111, 113).

## γδT in Psoriasis

Dysregulation of the immune system and T cell activation has been well demonstrated to play an essential role in psoriasis development. Several studies have attributed T cell function in the skin to αβT cells, while γδT cells have been often overlooked. IFN-γ-producing T helper (Th) 1 cells were initially thought to be primary drivers of psoriasis. However, substantial clinical and basic research findings in the past decade have proved that the interleukin (IL)-23/Th17 axis plays an important role in the pathogenesis of psoriasis (114, 115). Psoriatic inflammation was found to be impaired in IL-23- and IL-17-deficient mice, thereby confirming the involvement of the IL-23/IL-17 axis (116, 117). Th17 cells and their downstream effector molecules, including IL-17A, IL-17F, IL-22, and tumor necrosis factor (TNF-α), were found to be increased in the sera and psoriatic skin lesion (118). Recently, Th17 cells were found not to be the primary source of these pathogenic cytokines in psoriasis. Instead, IL-17A, IL-17F, and IL-22 were found to be produced by γδT cells (115). Injecting IL-23 into the skin of mice or applying a topical dose of imiquimod cream (5%) induced a typical psoriasis-like phenotype, i.e., epidermal thickness, erythema, and inflammation. These two models were demonstrated to mimic psoriasis-like inflammation and have been used to evaluate the efficacy of different treatment methods (119). Epidermal hyperplasia and inflammation response induced by IL-23/IMQ was observed to be significantly reduced in T cell receptor δ deficient (Tcrd^−/−^) mice, however, no significant changes were observed in T cell receptor β deficient Tcrb^−/−^ mice (120). In addition, Cai et al. demonstrated that upon IL-23 stimulation, IL-17 produced in Tcrd^−/−^ mice was significantly lower compared to WT or Tcra^−/−^ mice (121). These data further suggested that dermal γδT cells were the major IL-17-producing cells in the skin in response to IL-23 stimulation.

The production of IL-17 by dermal γδ T cells requires endogenous IL-1β (121). Mechanistically, IL-1β activates the mammalian target of rapamycin (mTOR) signaling pathway *via* IL-1R-MyD88, whereas IL-23 activates the STAT3 pathway. Transcription factor IRF-4 links the IL-1R and IL-23R pathways to induce enhanced IL-17 production in dermal γδ T cells (122). Both Vγ4 and Vγ6 dermal T cells produce IL-17, however, dermal Vγ4 T cells expand and produce significantly more IL-17 compared to Vγ6 (123). Dermal Vγ4 and Vγ6T cells have different effector signaling requirements. Dermal Vγ4 T cell proliferation and IL-17 production are dependent on STAT3, whereas dermal Vγ6 T cells may be activated through the STAT3-independent RelA/NF-kB pathway (122). Thus, dermal Vγ4 T cells appear to have a critical role in IMQ-induced psoriasis-like dermatitis (123).

Dermal γδT cells constitutively express IL-23R, IL-17R, RORγt, and the chemokine receptors CCR1, CCR2, CCR4, CCR5, CCR6, CXCR3, and CXCR4 (120, 121). CCL20, which is a unique CCR6 ligand, mediates skin infiltration of IL-17-producing γδT-cells and DCs. Numerous studies have shown that CCL20/CCR6 regulates T migration from the dermis to the epidermis, promotes neutrophil aggregation, and exacerbates inflammation (124). In IL-23-injected WT mice, CCL20 was highly upregulated with numerous CCR6+γδT cells observed in the epidermis (125). Anti-CCL20-neutralizing antibodies or engineered CCL20 variants with minimal chemotactic activity prevented the infiltration of IL-17-producing γδT-cell into the skin of IL-23-injected mice. This lead to IL-17 and IL-22 downregulation, blocked γδT cell recruitment to the epidermis, and reduced psoriasiform dermatitis (126, 127). In CCR6-knockout (KO) mice, γδT cells failed to migrate and accumulate in the epidermis after IL-23 treatment. Keratinocytes secrete CCL20, bind and activate CCR6, and regulate the migration of γδT cell subsets into the skin. This suggests the potential relevance of CCR6/CCL20 as a therapeutic target for psoriasis (126, 128, 129).

Psoriasis recurs frequently and relapse occurs in the same area after treatment discontinuation. Hence, recurrent psoriasis is a major problem that needs to be solved. TNF-a, IL-12/23, and IL-17 inhibitors have been shown to exhibit potent and rapid therapeutic efficacy (130, 131). However, these biological agents have been associated with several adverse events, the most common being susceptibility to infections (130). In addition to infections, biological inhibitors have been associated with demyelinating diseases, nasopharyngitis, upper respiratory infection, headaches, lupus, or lupus-like syndromes, mucocutaneous candidiasis, mild neutropenia, and new-onset or worsening of heart failure. The long-term safety concerns and high cost hamper the extensive use of these agents (130, 132, 133).

Psoriasis relapses around the original lesion area suggest these manifestations have an “immune memory.” Adaptive immune responses by memory T cells are not limited to foreign antigens, and relapses in autoimmune diseases are typically driven by auto-aggressive memory lymphocytes. There have been published reports regarding the adaptive-type memory responses in γδT cells. The response of human Vγ9Vδ2+ T cells to phospho-antigens is increased after initial *Mycobacterium bovis* BCG vaccinations (134). In macaques, a memory-type response and rapid expansion of Vγ9Vδ2 T cells have been observed after a secondary challenge with *Bacillus Calmette-Guerin* (135). Mouse “memory-like” Vγ6+ γδT cells were found to be retained for more than five months in the mesenteric lymph nodes after *Listeria monocytogenes* infection (136).

**Figure 2:**
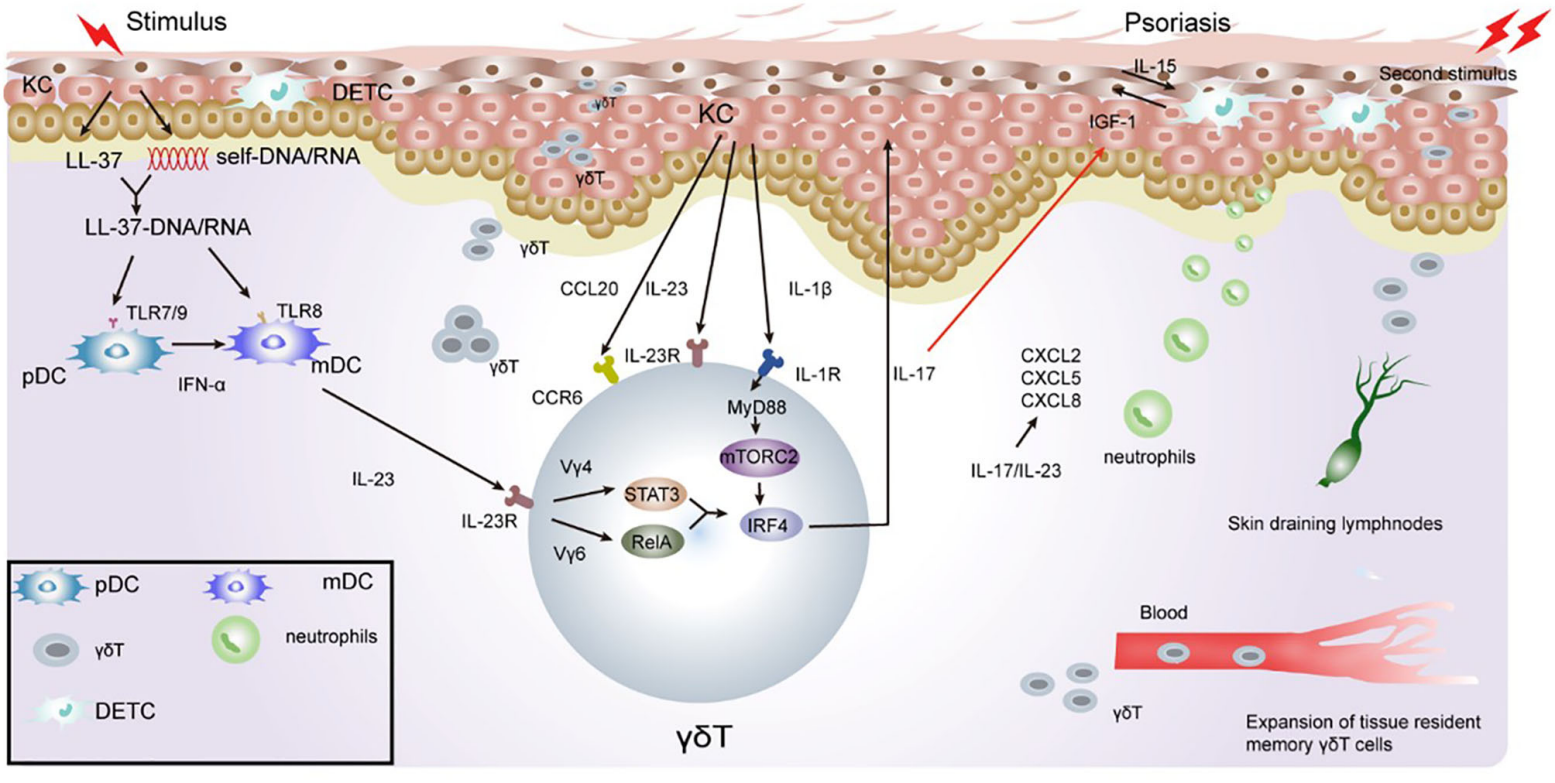
Role of γδT cells in the immune pathogenesis of psoriasis. Keratinocytes in the epidermis undergo apoptosis, necrosis, or death when exposed to certain external stimulation. With the release of cell contents, such as DNA and RNA, keratinocytes release antimicrobial peptides, such as LL-37. LL-37 binds with DNA and RNA to form a complex, promote immature DC activation, and secrete IFN-γ/IL-23 through the TLR7/8/9 pathway. IL-23 activates RORγt+γδT cells to secrete IL-17. γδT cell-derived IL-17 directly inhibits IGF-1 production in DETCs by increasing epidermal IL-23/IL-1β expression. During excessive keratinocyte proliferation, the secretion of TNF-α and chemokine ligand 20 (CCL20) increases, which consequently recruits CCR6+γδT cells to the inflammatory site of the epidermis. IL-17 cytokines produced by γδT cells potently upregulate the chemokine, CCL20, in keratinocytes, which chemoattracts IL-17A-producing CCR6+ immune cells to the inflamed site, thus forming a positive feedback loop. Il-23/IL-17 also promotes the recruitment of neutrophils to inflammatory sites, leading to excessive proliferation of the stratum corneum to form psoriatic inflammatory lesions. γδT cells have memory properties and can migrate rapidly to inflammatory sites through the blood and skin when subjected to a secondary stimulation. This consequently gives rise to severe inflammatory manifestations.

Memory-like γδT has been seen in psoriasiform mouse model, IL-17A-producing Vγ2Vδ4+ T cells initially derive from the neonatal thymus where they are instructed with tissue tropism. These Vγ2Vδ4+ T cells were phenotypically memory-like with a CD44hi CD62Llo CD27- expression pattern (137). After exposure to IMQ, Vγ4+γδT17 cells in the skin have been shown to rapidly expand in the draining lymph nodes (LNs) and then release from the LNs. They then migrate *via* the action of the chemokine, CCR2, to accumulate at sites of both inflamed and uninflamed skin in a S1P1-dependent manner. This in turn exacerbates the inflammatory response and recruitment of neutrophils. They have also been shown to migrate *via* the blood and persist in normal skin and peripheral LNs for a minimum of three months. Importantly, when subjected to the same second challenge at a distant skin site, memory-like Vγ4+γδT17 cells expand at a faster rate and produce more IL-17 compared to that after exposure to the first challenge, leading to a rapid and severe skin inflammatory response (19) (**Figure 2**). Sensitized mice showed elevated skin inflammation, significant cell proliferation, and IL-17 production by Vγ4+γδT cells upon IMQ challenge. Adoptive transfer experiments have confirmed that memory-like Vγ4+γδT17 cells respond rapidly, and their memory drives their involvement in the psoriasis recurrence (19, 138, 139).

γδT cells are rarely found in healthy human skin (140), however, they are easily generated from the skin of psoriatic patients. γδT cells have different adhesion properties compared to αβT cell subsets (141). A higher frequency of sequence sharing of the γ-chain has been found in psoriatic lesions from different individuals compared to those without psoriasis, suggesting that although the T cell response in psoriasis is highly polyclonal, particular γδT cell subsets could be associated with this disease (142). Following study demonstrated that an increased level of Vγ9Vδ2 T cells was present in psoriatic skin compared to healthy controls, while a significant reduction in Vγ9Vδ2 cells was observed in the blood of psoriasis patients. The number of circulating Vγ9Vδ2 T cells returned to normal levels after successful psoriasis-targeted treatment. These findings demonstrated the redistribution of Vγ9Vδ2 T cells from the blood to the skin of psoriasis patients (101). The recruitment of specific monoclonal population of γδT cells to psoriatic skin suggests local expression or modification of a cognate TCR ligand that is recognized by this population of memory-like γδT cells (143). Consistently, Zheng group found the higher expression of Vγ9 in psoriasis lesion than that in healthy individuals, indicating that Vγ9 γδT cells may be the main pathogenic cell (144). Additionally, Vγ9Vδ2 T cells have been shown to produce psoriasis-relevant cytokines, such as IFN-γ, TNF-α, and IL-17A and chemokines such as IL-8, CCL3, CCL4, CCL5, and CCR6. These cytokines and chemokines are responsible for recruiting crucial immune effector cells to the skin to activate keratinocytes (63, 145).

## Targeting γδT Cells for Psoriasis Therapy

The important role of dermal immobilized γδT cells in the pathogenesis of psoriasis has been elucidated in the past years. Hence, dermal γδT cells and their associated molecules have become attractive targets for drug development. Adiponectin, a metabolic mediator of insulin sensitivity, plays a crucial role in metabolic regulation and inflammatory/anti-inflammatory processes. Studies have demonstrated that in psoriasiform skin, inflammation, and infiltration of dermal γδT cells producing IL-17 were significantly enhanced in the absence of adiponectin. The negative regulation of adiponectin on IL-17 production from dermal γδT cells is mainly mediated through AdipoR1. This suggests that increasing adiponectin levels may be effective for improving psoriasis as well as metabolic disorders (146, 147). BTLA belongs to the immunoglobulin superfamily and has been reported to play a role in the homeostasis of γδT cells/ILCs in lymphoid tissues and controls the production of IL-17 in mature lymph node γδT cells. BTLA-deficient animal models have been shown to have a dysregulated proportion of inflammatory γδT cells and were susceptible to psoriasis and severe skin inflammation. BTLA agonism was found to limit the progression of these phenotypes. Activation of BTLA may restore the balance of γδT cell subsets to control autoimmune pathogenesis (148, 149). The agonistic anti-BTLA antibody (clone 6A6) was demonstrated to suppress γδT cell expansion and IL-17 production within the lymph nodes and skin induced by IMQ (149, 150). Thus, BTLA may be a potential target for the treatment of psoriasis. Dermal γδT cells constitutively express CCR6. CCR6KO or anti-CCL20 monoclonal antibodies administered to mice resulted in a decline in psoriatic dermatitis in IL-23-induced skin inflammation mouse models. This demonstrates that CCL20, together with its receptor, CCR6, are potential targets for the treatment of psoriasis (129, 151). CCL20 S64C is a CCL20 variant that binds to CCR6 and inhibits CCR6-mediated T cell migration. Previous studies have shown that CCL20 S64C alleviates the inflammatory response in psoriasis-like models induced by IL-23, and have been associated with reduced accumulation of CCR6+ IL-17-producing γδT cells in the epidermis (127). FTY720 is an FDA-approved immunomodulatory drug for the treatment of multiple sclerosis. It reduces lymphocyte egress from lymphoid tissues by inhibiting the sphingosine-1 phosphate receptor (S1PR). FTY720 inhibits the migration of Vγ4+VγT4+ T17 cells from the lymph nodes to the skin, suggesting its potential as a treatment for psoriasis (152). Indirubin (IR) is a bisindole compound extracted from the leaves of the Chinese herb *Indigo naturalis*. It has been demonstrated to alleviate IMQ-induced psoriasis-like dermatitis by primarily reducing the inflammatory responses mediated by IL-17 A-producing γδT cells through Jak3/Stat3 activation (153). Dashlkhumbe et al. reported a newly formulated methotrexate (MTX, a chemical conjugate of MTX with a cell-permeable peptide) for the treatment of psoriasis. Topically applied skin-penetrating (SP)-MTX reduced the psoriasiform skin phenomenon and epidermal thickness by reducing CD11c+, CD4+, and IL-17-producing γδT cell-containing infiltrate of immune cells in the skin (154).

## Conclusions and Future Directions

Psoriasis has a complex and varied pathogenesis. During disease development, γδT cells secrete proinflammatory cytokines, such as IL-17 and IFN-γ, which induce and aggravate psoriasis. Notably, γδT cells have memory cell properties that rapidly respond to secondary stimulation. This contributes to the recurrence of psoriasis.

Future studies should investigate whether γδT cells that reside in skin lesions have resident memory cell properties, how long they persist, how often they turn over, and what environmental niches within peripheral tissues support their long-term survival. Studies have shown that metabolism and immune function are tightly linked (155, 156). Nutrient availability and cellular metabolism tightly control the differentiation, survival, and function of immune cells (157). However, whether cellular metabolism regulates γδT fate decisions remains to be deciphered. Additional studies are necessary to identify the mechanisms that reduce γδT cells to prevent the recurrence of psoriasis.

## Author Contributions 

CQ drafted and edited the manuscript. CQ drafted and edited the figures and figure legends. YW and PL edited the manuscript. JZ edited and approved the final version of the manuscript. All authors contributed to the article and approved the submitted version.

## Funding

This work was supported by the National Natural Science Foundation of China (No. 81673989 and No. 82074434) and the Beijing municipal health system high-level health technology talent team construction project (No. 2015-3-116).

## Conflict of Interest

The authors declare that the research was conducted in the absence of any commercial or financial relationships that could be construed as a potential conflict of interest.
